# White matter changes following electroconvulsive therapy for depression: a multicenter ComBat harmonization approach

**DOI:** 10.1038/s41398-022-02284-3

**Published:** 2022-12-16

**Authors:** Jean-Baptiste Belge, Peter C. R. Mulders, Linda Van Diermen, Didier Schrijvers, Bernard Sabbe, Pascal Sienaert, Mardien L. Oudega, Indira Tendolkar, Annemieke Dols, Philip van Eijndhoven

**Affiliations:** 1 Department of Psychiatry, Collaborative Antwerp Psychiatric Research Institute (CAPRI), Faculty of Medicine and Health Sciences, University of Antwerp, Campus Drie Eiken, S.033, Universiteitsplein 1, 2610 Wilrijk, Belgium; 2 Department of Psychiatry, Radboud University Medical Centre, P.O. Box 9101, 6500 HB Nijmegen, The Netherlands; 3 Donders Institute for Brain, Cognition and Behavior, Centre for Medical Neuroscience, P.O. Box 9010, 6500 GL Nijmegen, The Netherlands; 4 Psychiatric Center Bethanië, Andreas Vesaliuslaan 39, 2980 Zoersel, Belgium; 5 Department of Psychiatry, University Psychiatric Center Duffel, Stationstraat 22, 2570 Duffel, Belgium; 6 University Psychiatric Center KU Leuven, Academic Center for ECT and Neuromodulation (AcCENT), Leuvensesteenweg 517, 3010 Kortenberg, Belgium; 7 Department of Old Age Psychiatry, GGZ inGeest Specialized Mental Health Care, Oldenaller 1, 1081 HJ Amsterdam, the Netherlands; 8 Amsterdam UMC, Vrije Universiteit, Psychiatry, Neuroscience, Amsterdam, the Netherlands

**Keywords:** Depression, Psychiatric disorders

## Abstract

ECT is proposed to exert a therapeutic effect on WM microstructure, but the limited power of previous studies made it difficult to highlight consistent patterns of change in diffusion metrics. We initiated a multicenter analysis and sought to address whether changes in WM microstructure occur following ECT. Diffusion tensor imaging (DTI) data (*n* = 58) from 4 different sites were harmonized before pooling them by using ComBat, a batch-effect correction tool that removes inter-site technical variability, preserves inter-site biological variability, and maximizes statistical power. Downstream statistical analyses aimed to quantify changes in Fractional Anisotropy (FA), Mean Diffusivity (MD), Radial Diffusivity (RD) and Axial Diffusivity (AD), by employing whole-brain, tract-based spatial statistics (TBSS). ECT increased FA in the right splenium of the corpus callosum and the left cortico-spinal tract. AD in the left superior longitudinal fasciculus and the right inferior fronto-occipital fasciculus was raised. Increases in MD and RD could be observed in overlapping white matter structures of both hemispheres. At baseline, responders showed significantly smaller FA values in the left forceps major and smaller AD values in the right uncinate fasciculus compared with non-responders. By harmonizing multicenter data, we demonstrate that ECT modulates altered WM microstructure in important brain circuits that are implicated in the pathophysiology of depression. Furthermore, responders appear to present a more decreased WM integrity at baseline which could point toward a specific subtype of patients, characterized by a more altered neuroplasticity, who are especially sensitive to the potent neuroplastic effects of ECT.

## Introduction

Advances in neuroscience have highlighted depression as a syndrome caused by aberrant interactions among various networks in the brain [1]. Neuroimaging research in patients with depression, highlights functional and structural alterations in and between the components of a complex limbic-cortical-striatal-pallidal-thalamic circuit [1, 2]. Importantly, both the structural connectivity, and the strength and persistence of functional connectivity between these different nodes of the brain are underpinned by the architectural and microstructural properties of the white matter (WM) [3, 4]. Diffusion tensor imaging (DTI) measures the restriction of freely moving water molecules in brain tissue [5] and metrics like fractional anisotropy (FA), mean (MD), radial (RD) and axial (AD) diffusivity describe WM microstructural properties [6, 7]. Of these metrics, FA has been most commonly used to describe changes in the WM microstructure across various neuropsychiatric disorders, with higher FA values thought to represent increased WM integrity [5–7]. Research employing DTI, consistently reports alterations of WM microstructure in patients with depression [5]. Most of these investigations point toward reductions of FA in frontal, limbic and striatal connections, including lower FA in the anterior limb of the internal capsule, inferior longitudinal fasciculus, posterior thalamic radiation, the superior longitudinal fasciculus, and the fronto-occipital fasciculus [5]. More pronounced reductions were found to be related to insufficient treatment response [8]. Recently, the major depressive disorder working group of the Neuroimaging Genetics through Meta-Analysis (ENIGMA) consortium confirmed these alterations through an analysis across 20 international cohorts [5]. They observed subtle, but widespread, lower FA in patients compared with controls with the largest differences being observed in the corpus callosum and corona radiata, suggesting structural dysconnectivity in patients with depression [5]. Interestingly, ECT, the most effective biological treatment for depression [9] seems to exert an influence on WM microstructural properties and architecture [10–16]. It has been proposed that these effects rely on ECT’s potent neuroplastic properties, which may in case of the WM also specifically depend on the seizures that are propagated along the WM tracts in the brain [12, 16]. Whilst sparsely investigated, ECT induces indeed changes in various diffusion metrics [10–16]. In a pilot study Nobuhara et al. [14], (*n* = 8) reported an increase in FA after ECT in frontal WM [14]. In a subsequent study, Lyden et al. (2014) (*n* = 20) observed that FA increased in the anterior cingulum, forceps minor, and left superior longitudinal fasciculus [12]. This increase was moreover positively associated with treatment response. However, Nickl-Jockschat et al. (2016) (*n* = 21) did not detect any WM alterations associated with ECT [13]. Finally, in the most recent study, Gryglewski et al. [11] (*n* = 13) observed an increase in AD, an indirect measure of axonal integrity, in the posterior limb of the internal capsule of the right hemisphere but changes of FA were not observed [11]. The inconsistency of these results could be due to the limited power and methodological differences between these studies, which makes it difficult to highlight consistent patterns of change in diffusion metrics following ECT. Further, the clinical relevance of the observed changes remains unclear. A better understanding of the impact of ECT on WM microstructure could further our understanding of the neurobiological underpinnings of the potent therapeutic effects of ECT. In the current DTI project, we aimed to increase statistical power and overcome methodological differences between individual studies by conducting a multicenter analysis on existing cohorts to perform the largest analysis on WM changes following ECT in patients with depression to date. We harmonized DTI data from the different sites before pooling them by using the novel analysis technique, Combat, which corrects for inter-site technical variability while preserving inter-site biological variability and maximal statistical power [17]. In our downstream statistical analyses, we first aimed to quantify longitudinal changes before vs after ECT in FA, MD, RD and AD after ECT using whole-brain, tract-based spatial statistics (TBSS). Second, we investigated whether the observed changes could be linked to changes in depressive symptoms as a result of ECT. Finally, Cross-sectional analyses were also carried out to investigate differences in WM integrity between responders and non-responders at baseline.

## Materials and methods

### Participants

For this multicenter study, 4 different cohorts were combined. Sample I consisted of 17 patients recruited at the University Psychiatric Center (UPC) in Duffel, Belgium. Sample II consisted of 13 patients recruited at the GGZ in Geest, Amsterdam, the Netherlands. Sample III and IV were both recruited at the department of Psychiatry of the Radboud University Medical Centre, Nijmegen, the Netherlands, and consisted of 17 and 11 patients respectively. For all samples, patients needed to fulfil the following inclusion criteria: a diagnosis of unipolar depression as defined by the DSM-IV-TR criteria (to note, in sample I, patients with a diagnosis of bipolar depression were also included (*n* = 3)), ECT treatment in accordance with the Dutch Guidelines on Electroconvulsive Therapy [18] and the presence of two good quality DTI data sets (before and after ECT) per patient. Exclusion criteria were drug or alcohol dependence, a primary psychotic disorder as assessed using the MINI interview [19], and contraindications for MRI (e.g., a pacemaker, claustrophobia, metallic implants). In addition, patients with a major neurological illness, including Parkinson disease, stroke, and dementia, were excluded. In total, 58 participants were enrolled from whom written informed consent was obtained. All procedures were approved, for sample I, by both the local Ethics Board of the UPC Duffel and the central ethics committee of the University Hospital Antwerp, for sample II, by the Ethical Review Board of the Amsterdam University Medical Centre, and for sample III and IV by the local ethics committee of the Radboud University Medical Centre. The authors assert that all procedures contributing to this work comply with the ethical standards of the relevant national and institutional committees on human experimentation and with the Helsinki Declaration of 1975, as revised in 2008.

### ECT procedure

ECT was administered twice weekly in accordance with recent guidelines (Broek et al. [18]) using a brief-pulse (0.5 ms (sample I)/1.0 ms (sample II–IV)) constant-current Thymatron IV system (Somatics LLC, Lake Bluff, IL, USA). The electrodes were placed unilaterally over the right hemisphere (RUL) or bitemporal (BT) when a fast antidepressant or antisuicidal effect was required or when patients did not respond to unilateral ECT [20]. To note, in sample III, all patients were treated with a BT electrode placement. Before the first session, the stimulus dose was determined using the age method for RUL treatment and the half-age method for the bilateral electrode placement in sample I [21]. In sample II–IV, the stimulus dose was determined using the ST (seizure threshold) titration method. After seizure threshold was determined at the first ECT session, subsequent treatments were delivered at energy settings 5× ST for right unilateral ECT and at 1.5× ST for bilateral ECT. Etomidate (0.15 mg/kg) was the anesthetic routinely used. Succinylcholine (succinylcholine, 0.5 mg/kg) was used as muscle relaxant. ECT was continued until the patient was either in remission (HDRS17 ≤ 7) or showed no further improvement during the last three sessions.

#### Clinical measures

All clinical assessments were conducted within 1 week before(T0) the start of the first ECT session and within 1 week after (T1) the last ECT session.

### Depressive symptoms

Depressive symptoms were assessed using the Hamilton Rating Scale for Depression–17 items (HDRS-17) [22]. Treatment response was defined as a reduction of 50% or more on the HDRS17. In sample II, depressive symptoms were assessed by the Montgomery and Asberg Depression Rating scale, but scores were converted to HDRS-17 equivalents employing adequate formulae [23].

### MRI acquisition and processing

#### DTI data acquisition

For this multicenter DTI study, DTI scans were collected at three different sites employing four different scanners constituting as such 4 different samples: (I) the UPC Duffel sample (II), the Amsterdam UMC sample, (III) the Radboud UMC-1 sample, (IV) the Radboud UMC-2 sample. For each sample, a single-shot, diffusion-weighted, echo planar imaging sequence was consistently obtained within the week before a participant’s first ECT session (T0) and within 1 week (T1) after completion of the acute course. Scanners and Acquisition parameters for each site were as followed: (I) A 3 T Siemens Magnetom Prisma MRI scanner (Erlangen, Germany): TE, 71 milliseconds; TR, 8500 milliseconds; image resolution: 2.0 mm isotropic; b value, 1000 s/mm2; 30 directions; 75 axial slices. (II) A General Electrics Sigma HDxt 3 T scanner (General Electric, Milwaukee, WI, USA); TE, 76 milliseconds; TR, 7150 milliseconds; image resolution: 2.4 mm isotropic; b value, 1000 s/mm2; 30 directions; 60 axial slices. (III) A 1.5 T Siemens Avanto system (Erlangen, Germany): TE, 85 milliseconds; TR, 7400 milliseconds; image resolution: 1.6 mm isotropic; b value, 1000 s/mm2; 34 directions; 75 axial slices. (IV) A 3 T Siemens Magnetom Prisma MRI scanner (Erlangen, Germany): TE, 76 milliseconds; TR, 7200 milliseconds; image resolution: 2.0 mm isotropic; b value, 1000 s/mm2; 75 axial slices; 30 directions.

### DTI preprocessing

Preprocessing and statistical analyses were performed on all pre- and post-ECT DTI scans collected using FSL 6.0.3 (FMRIB, Oxford, UK). For each subject, diffusion-weighted images were corrected for motion and eddy-current distortions using the eddy commando with the b0 image as reference for alignment [24]. After eddy-current correction a diffusion tensor model was fitted at each voxel using DTIFIT. The resultant eigen values were used to compute FA, AD (λ1), RD ((λ2 + λ3)/2), and MD.

### DTI harmonization

The ComBat harmonization method, as implemented in MATLAB was used after imaging processing and before downstream statistical analyses (TBSS) to remove inter-site scanner-related technical variability while preserving inter-site biological variability and maximize statistical power [17]. To note the ComBat harmonization method (https://github.com/Jfortin1/ComBatHarmonization) is an empirical Bayesian method for data harmonization that was originally designed for genomics [17]. ComBat has been validated by various other studies, in structural but also functional and volumetric modalities [17, 25, 26]. Moreover, the ENIGMA working group recommends applying the ComBat algorithm to attenuate potential effects of site in multi-site structural imaging work [27]. As a matter of fact, in comparison with other available harmonization methods, ComBat performs best at modeling and removing the unwanted inter-site variability in diffusion parameters whilst preserving biological variability and maximizing statistical power [17]. For this study, age and sex were used as biological covariates of interest.

### Statistical analyses

Voxel-wise statistical analysis of the harmonized diffusion data (FA, MD, AD, and RD) was carried out using TBSS (Tract-Based Spatial Statistics, [28] implemented in FSL [29]. FA images were first aligned within and across subjects and then to standard MNI152 space using combined nonlinear and affine registrations. A mean FA image was subsequently created to represent a FA skeleton common to all subjects and time points. The aligned FA data from each subject or time point were then projected onto this skeleton to allow voxel-wise comparisons. The transformation files and skeleton projection vectors generated for the FA images were applied to the MD, RD and AD images to allow comparison of these diffusion metrics in the same common space. FSL’s Randomize tool (http://www.fmrib.ox.ac.uk/fsl/randomise/index.html), which combines the general linear model with permutation testing, was then used for voxel-based analysis of each diffusion metric. The anatomical locations of clusters showing significant effects were identified using the Johns Hopkins University DTI-based WM atlas [30]. First, paired *t*-tests established longitudinal WM changes in FA, MD, RD and AD before (T0) vs after (T1) ECT. To assess the possible influence of ECT laterality and number of ECT sessions on those WM changes reaching statistical criterion following ECT, we carried out an analysis of covariance (ANCOVA), with change as the dependent variable and number of ECT sessions and electrode position as independent variables. Second, we investigated whether the observed changes could be linked to changes in depressive symptoms as a result of ECT. To do this difference scores (T0–T1) were computed for the HDRS-17 scores and a paired *t*-test was carried out. Exploratory Pearson correlations between the significant changes (T0–T1) in diffusion metrics and changes in HDRS-17 scores (T0–T1) were then performed. To assess the possible associations between change in diffusion metrics and changes in HDRS scores at a whole brain level, we also performed a GLM with the change in HDRS scores as regressor. Bonferroni correction was employed for the correlational analysis with a *p* value fixed at 0.004 (0.05/number of regions presenting a significant longitudinal change in diffusion parameters (*n* = 12)). To assess on a whole brain-level the relationship between clinical response and changes in DTI metrics we performed a cross-sectional comparison in WM change between responders and non-responders. Further, a cross-sectional analysis in WM properties between responders and non-responders at baseline (T0) was carried out.

WM changes were considered significant using an FDR corrected *p* value of 0.05. All statistical analyses were performed using JMP SAS version 14-PRO.

## Results

### Participants

Fifty-eight DTI datasets were obtained (43 females and 15 males, mean age = 56.36) both before and after ECT. Demographics, clinical characteristics, and ECT treatment information are presented in Table 1. ECT had a significant effect on depressive symptoms, as is demonstrated by the significant drop in HDRS-17 scores (*p* < 0.0001, *t* = −9.52). The response rate to ECT was 62.3%. Responders and non-responders did not differ on baseline demographic variables.

**Table 1:** Demographics, clinical characteristics, and ECT protocol information.

Variable	*n* = 58
Demographic Information	
Age, mean years (SD)	56.36 (12.20)
Gender (M/F)	15/45
Clinical Characteristics	
Current episode duration, mean months (SD)	20.53
Unipolar/bipolar depression (n)	55/3
ECT Protocol	
Unilateral electrode placement	26
Bilateral electrode placement	19
Electrode switch (unilateral -> bilateral)	13
Number of ECT Index sessions, mean (SD)	13.95
Number of ECT Index sessions, range	8.89
Responders^a^ *n* (%)	33 (62.27%)

*ECT* electroconvulsive therapy, *F* female, *M* male, *SD* standard deviation, *HDRS17* Hamilton Depression Rating Scale 17 Items.

^a^Response was defined as a 50% or larger baseline to end-of-treatment reduction in HDRS17 scores. Response data were only available for *n* = 53 patients.

### Longitudinal effects of ECT on diffusion metrics (FA, AD, RD, MD)

**Fig. 1:**
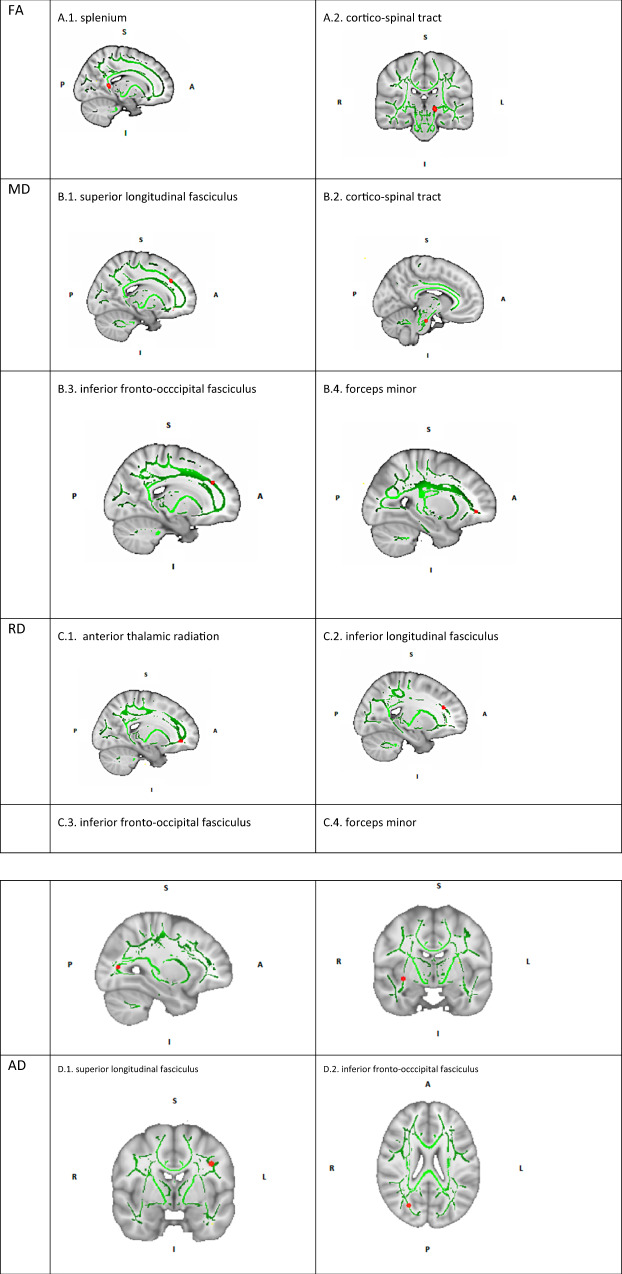
Longitudinal changes of diffusivity metrics following ECT. An increase of FA was observed in the right splenium of the corpus callosum (**A.1**.) and the left cortico-spinal tract (**A.2**.). An increase in AD was observed in the left superior longitudinal fasciculus (**D.1**.) and in the right inferior fronto-occipital fasciculus (**D.2**.). MD did increase in the left superior longitudinal fasciculus (**B.1**.), the left cortico-spinal tract (**B.2**.) and the left inferior fronto-occipital fasciculus (**B.3**.). RD did increase in the right anterior thalamic radiation (**C.1**.), the left inferior longitudinal fasciculus (**C.2**.) and the right inferior fronto-occipital fasciculus (**C.3**.). The right forceps minor showed both an increase in MD and RD (**B.4., C.4**.). Abbreviations: FA = fractial anisotropy, MD = mean diffusivity, RD = radial diffusivity, AD = axial diffusivity, I = inferior, S = superior, R = right, L = left, A = anterior, P = posterior.

After FDR correction, only increases in diffusion metrics remained significant. After ECT, a significant increase of FA was observed in the right splenium of the corpus callosum and the left cortico-spinal tract. Furthermore, patients showed significant increases in AD in the left superior longitudinal fasciculus and in the right inferior fronto-occipital fasciculus. Increases in MD and RD could be observed in overlapping WM structures of both hemispheres. MD did increase in the left superior longitudinal fasciculus, the left cortico-spinal tract and the left inferior fronto-occipital fasciculus. RD did increase in the right anterior thalamic radiation, the left inferior longitudinal fasciculus and the right inferior fronto-occipital fasciculus. Finally, the right forceps minor showed both an increase in MD and RD (See Table 2 and Fig. 1). The observed WM changes were not influenced by the number of ECT sessions or electrode position. It should be noted that a negative interaction (*p* = 0.0169) was observed between the number of ECT sessions and an increase of MD in the left cortico-spinal tract, this interaction failed however to remain significant after Bonferroni correction.

**Table 2:** Longitudinal and cross-sectional analysis of diffusion metrics.

Diffusion metrics	Effect	Brain areas	Hemisphere	Max. Z (X, Y, Z)	*p* value	*t*-value
LONGITUDINAL						
Fractial Anisotropy						
	↑	Cortico-spinal tract	Left	105,102,68	0.0372	3.68
	↑	Splenium	Right	73,83,82	0.007741	2.70
Mean Difusivity						
	↑	Superior longitudinal fasciculus	Left	107, 156, 105	0.00321	2.94
	↑	Corticospinal tract	Left	98,106,47	0.0196	2.65
	↑	Forceps Minor	Right	71,161,103	0.0174	3.84
	↑	Inferior Fronto-occipital fasciculus	Left	116,170,67	0.000335	2.94
Radial Diffusivity						
	↑	anterior thalamic radiation	Right	70, 169, 67	0.0155	2.30
	↑	Forceps minor	Left	111,160,93	0.0126	2.35
	↑	Inferior Longitudinal fasciculus	Left	119,52,80	0.03583	2.48
	↑	Inferior fronto-occipital fasciculus	Right	55,118,65	0,007375	2.48
Axial Diffusivity						
	↑	superior longitudinal fasciculus	Left	130,123,105	0.006	1.89
	↑	Inferior fronto-occipital fasciculus	Right	63,56,95	0.01946	2.41
CROSS-SECTIONAL (T0; R vs NR)						
Fractial Anisotropy	↓	Forceps Major	Left	106,38,85	0.028	−2.53
Mean Diffusivity	**-**	**-**	**-**	**-**	**-**	
Radial Diffusivity	**-**	**-**	**-**	**-**	**-**	
Axial Diffusivity	↓	Uncinate Fasciculus	Right	50,132,45	0.004	−2.36

*T0* baseline, *R* responders, *NR* non-responders.

### Cross-sectional effects between responders and non-responders at baseline (T0)

**Fig. 2:**
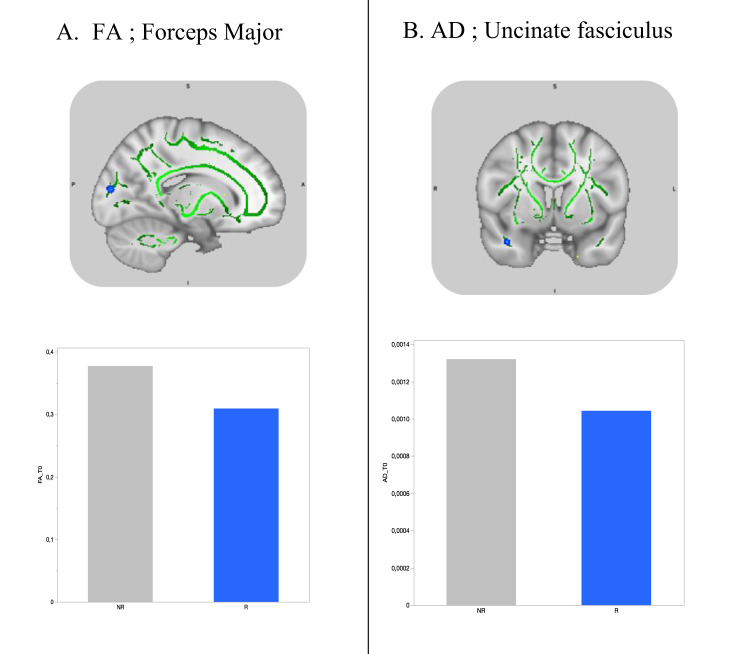
Cross-sectional effects between responders and non-responders at baseline. **A** Responders compared to non-responders showed significantly smaller FA values in the in the left forceps major at baseline. **B** Responders compared to non-responders showed significantly smaller AD values in the right uncinate fasciculus at baseline. FA = fractial anisotropy, AD = axial diffusivity, I = inferior, S = superior, R = right, L = left, A = anterior, P = posterior, T0 = baseline, R = responders, NR = non-responders.

After FDR correction, analyses revealed appreciable differences between the responder and non-responder group at baseline (T0). Specifically, responders showed significantly smaller FA values in the left forceps major and smaller AD values in the right uncinate fasciculus compared with non-responders. No differences between groups were observed for the RD and MD metrics (See Table 2. and Fig. 2).

### Cross-sectional comparison in WM change between responders and non-responders

No significant differences in change of diffusivity metrics between responders and non-responders were observed.

### Exploratory correlations between ECT-induced changes in diffusion metrics and the clinical outcomes

No significant associations were observed between the ECT-induced changes in diffusion metrics and the changes in HDRS-17 scores. It should be noted that the observed positive correlation between the post-ECT change in MD of the left cortico-spinal tract and the HDRS-17 scores was driven by an outlier (Cook’s D > 3 times the mean), which was removed before analysis. Further, at the whole brain level no significant associations were found between changes in diffusion metrics and changes in HDRS scores.

## Discussion

### Longitudinal effects of ECT on FA

Employing a harmonization approach we are the first to observe an increase in FA in the splenium following ECT. This brain region is the most posterior part of the corpus callosum, composed of reciprocal fibers from the temporal association and parietal association, connecting regions of the parietal and occipital lobes through the forceps major [31, 32].

Reductions of FA are consistently reported in the corpus callosum of patients with depression [5, 33–36] and a decrease of myelin in the axons of the splenium has been demonstrated in a post-mortem sample of patients with depression [37]. The splenium is known to synchronize interhemispheric oscillatory activity through the balanced excitation or suppression of the contralateral homotopic and heterotopic cortical areas [38]. This is important as depression is related to interhemispheric functional synchronization deficits [39–42]. Interestingly, ECT has been shown to selectively modulate this interhemispheric functional synchronization [43]. As increased FA suggests increased fiber coherence and organization [7], our results could suggest that ECT restores interhemispheric connectivity through its neuroplastic properties on splenial WM and its efferent fiber tracts. Of note, also the left cortico-spinal tract showed an increased FA. Psychomotor retardation, a core symptom of depression, seems to be addressed by cortico-spinal connectivity, with higher FA values reflecting a compensatory mechanism in depressed patients [44]. With the potent impact of ECT on psychomotor symptoms in mind [45] future studies should further investigate the modulation of this pathway to comprehend the neurobiology behind these effects.

### Longitudinal effects of ECT on AD

Our post-ECT analyses revealed an increase of AD in the left superior longitudinal fasciculus and the right inferior fronto-occipital fasciculus. Altered integrity of these WM tracts has been frequently observed in depressed patients and is suggested to underpin fronto–subcortical dysconnectivity [33–36]. Some studies have related lower AD to axonal damage and fragmentation, with an increase of AD thought to represent the normalization of alterations in axonal integrity [46]. Our finding complements previous work of Lyden et al. [12] who observed an increase of WM integrity in the superior longitudinal fasciculus [12]. Interestingly, the superior longitudinal fasciculus participates in the modulation of the dorsolateral prefrontal cortex (DLPFC) [47–49]. WM dysconnectivity of this tract appears to drive an attenuated top-down cognitive control from the DLPFC of limbic hyperactivity, instigating ruminative or repetitive negative thinking, a core behavior of the depressive syndrome [49, 50]. In unison with Lyden et al. [12], it is thus tempting to propose that the microstructural changes of the superior longitudinal fasciculus by ECT could enhance top-down cognitive control over emotional states to regulate mood, constituting in essence a core mechanism behind the therapeutic effects of ECT [12]. This corroborates moreover the functional imaging findings of our group demonstrating an enhanced connectivity of the DLPFC following ECT [51].

### Longitudinal effects of ECT on MD and RD

We observed an increase of MD, encompassing the left superior longitudinal fasciculus, the left cortico-spinal tract, the left inferior fronto-occipital fasciculus and the right forceps minor. These findings are in line with both Repple et al. (2019) and Gryzelewski et al. (2020) but in contrast to the study of Lyden et al. [12], who observed a global decrease of MD [11, 12, 15]. To note, both the studies of Repple et al. (2019) and Gryzelewski et al. (2020) observed a right lateralization of the MD increase [11, 15]. This could reflect the fact that the vast majority of patients were stimulated unilaterally on the right side in these studies, whilst our patients received both right unilateral and BT stimulation. MD is a measure of the overall diffusivity in a particular voxel regardless of direction [6]. It has been proposed that the MD increase could reflect a moderate ECT-induced increase in water concentration due to an increased permeability of the blood-brain barrier [52]. Further, coinciding with Gryzelewski et al., (2020), the increases of MD were observed in the same regions showing FA and AD effects. Respectively in the left cortico-spinal tract and the left superior longitudinal fasciculus [11].

It is thought that during the transient increase of permeability of the blood-brain barrier certain neuroactive reactants such as the brain-derived neurotrophic factor (BDNF), an important neuroplastic agent, may get released from the circulation to the brain, promoting neuroplastic mechanisms [52, 53]. This could explain the overlap between increased MD and FA/AD, thought to reflect augmented WM integrity.

Similar to findings of Gryzelewski et al. (2020), we observed an increase of RD in the right anterior thalamic radiation, the left inferior longitudinal fasciculus, the right inferior fronto-occipital fasciculus, and the right forceps minor [11]. On the other hand, Lyden et al. [12], did observe a global decrease of RD, especially in those regions presenting an increase of FA [12]. Whilst we did not replicate these findings, we did however find an overlap with those regions presenting an increased AD, as was the case for Gryzelewski et al. (2020) [11]. Increases in RD may suggest WM de- or dys-myelination [6]. The fact that the observed increases in AD did not translate into an increase in FA could be attributed to the increase in RD [11].

### Cross-sectional effects between responders and non-responders at baseline

The identification of neuroimaging biomarkers of treatment response holds promise toward personalizing ECT and improving treatment outcomes. To our best knowledge, we are the first to demonstrate smaller FA values in the left forceps major and smaller AD values in the right uncinate fasciculus in responders compared to non-responders. Whereas the forceps major emerges from the splenium to connects regions of the parietal and occipital lobes [31, 32], the uncinate fasciculus connects inferior frontal regions with medial temporal regions, such as the amygdala and hippocampus and is thought to be important for the alterations of frontolimbic circuitry observed in depression [54]. Our findings suggest thus a more decreased WM integrity at baseline in the responder group.

The relationship between baseline neuroplasticity and treatment outcome following ECT has been sparsely investigated, but Neyazi et al., [55] found pre-ECT an increased p11 promoter methylation in treatment-responders, which could reflect a decreased gene expression of important neuroplastic agents such as BDNF in this group [55]. This could suggest that ECT responders do present a more altered neuroplasticity at baseline, which is in in line with the decreased FA values of our study. It is thus tempting, although also highly speculative, to propose based on our results and those of Neyazi et al. [55], that ECT responders could be constituted by a specific subgroup of patients, characterized by a more altered neuroplasticity at baseline, who are especially sensitive to and can benefit most optimally from the potent neuroplastic effects of ECT.

Beyond its longitudinal design, the main strength of our study resides in its harmonization approach, in which the imaging data are combined before performing statistical inferences, increasing the statistical power compared to meta-analyses. In addition, by pooling imaging data across sites our study enriched the clinical picture of the sample by increasing the variability in symptom profiles and demographic variables [17]. Moreover, by employing the state-of-the-art ComBat harmonization technique, we removed the unwanted variability introduced by site heterogeneity of the imaging measurements by differences in scanner protocols while preserving biological variability [17]. Further, the sensitivity of the TBSS approach to examine WM integrity using scalar measures such as FA, MD, RD and AD has also been well established across a number of neuropsychiatric disorders. Despite the robustness of our results, there are also some limitations to be considered. TBSS may indeed have some limitations to characterize all the voxels that are specific to a tract [56].

Further, the ComBat method assumes the site effect parameters to follow a particular parametric prior distribution, which might not generalize to all scenarios or measures, and it is not yet clear how non-linearities in the signal due to site effects propagate through the preprocessing techniques, as well as model fitting procedures [57]. It should also be noted that diffusion metrics are not a direct measure of WM integrity. Interpretation of these metrics should be done carefully as all that is proven is that there is a change in the diffusion parameters of water in a specific neural region, the interpretation of which is merely a plausible hypothesis [58].

The lack of a control dataset to confirm our findings should be mentioned. As a matter of fact, the specificity of these ECT related effects should be scrutinized by comparing them with a sample of depressed patients put on other forms of treatment. Further, differences in WM changes following ECT between treatment resistant and other forms of depression should also be addressed, as form specific alterations of structural brain characteristics have been suggested by previous research [59]. Finally, as we were not able to demonstrate a relationship between the observed changes in diffusivity metrics and therapeutic response, our results should be interpreted carefully and the proposed association with the therapeutic effects of ECT remain speculative and should be confirmed in larger samples.

## Conclusion

In summary, ours is the first multicenter analysis to investigate changes in diffusion metrics following ECT. Our data demonstrate that ECT normalizes altered WM microstructure in important brain circuits that are implicated in the pathophysiology of depression. Importantly, our findings parallel functional neuroimaging findings that ECT could enhance top-down cognitive control over emotional states to regulate mood. Furthermore, initial evidence indicates that ECT responders present a more altered WM integrity at baseline, indicating as such a subgroup of depressed patients, especially sensitive to ECT.
